# Epididymal Region-Specific miRNA Expression and DNA Methylation and Their Roles in Controlling Gene Expression in Rats

**DOI:** 10.1371/journal.pone.0124450

**Published:** 2015-04-22

**Authors:** Chen Chu, Guangyong Zheng, Shuanggang Hu, Jinsong Zhang, Shengsong Xie, Wubin Ma, Minjie Ni, Chunhua Tang, Lu Zhou, Yuchuan Zhou, Mofang Liu, Yixue Li, Yonglian Zhang

**Affiliations:** 1 State Key Laboratory of Molecular Biology, Shanghai Key Laboratory of Molecular Andrology, Institute of Biochemistry and Cell Biology, Shanghai Institutes for Biological Sciences, Chinese Academy of Sciences, 200031, Shanghai, China; 2 CAS-MPG Partner Institute for Computational Biology, Shanghai Institutes for Biological Sciences, Chinese Academy of Sciences, 200031, Shanghai, China; 3 Key Lab of Systems Biology, Shanghai Institutes for Biological Sciences, Chinese Academy of Sciences, 200031, Shanghai, China; 4 Graduate University of the Chinese Academy of Sciences, 200031, Shanghai, China; Clermont-Ferrand Univ., FRANCE

## Abstract

Region-specific gene expression is an intriguing feature of the mammalian epididymis. This unique property is essential for sperm maturation and storage, and it also implicates stringent and multi-level regulations of gene expression. Over the past decade, the androgen-driven activation of epididymal gene transcription has been extensively studied. However, it still remains largely unexplored whether and how other regulatory mechanisms, such as miRNAs and DNA methylation, are involved in controlling regional gene expression in the epididymis. Using microarray-based approaches, we studied the regional miRNA expression and DNA methylation profiles in 4 distinct epididymal regions (initial segment, caput, corpus and cauda) of rats. We found that the miR-200 family members were more expressed in caput, compared with cauda. By GSEA analysis, the differential expression of miR-200 family between caput and cauda was shown to be negatively correlated with their predicted target genes, among which 4 *bona fide* targets were verified by luciferase reporter assay. Predicted target genes of miR-200 family have enriched functions in anti-apoptosis, cell transportation and development, implying the regional diversity in epididymal functions. On the other hand, we revealed epididymal DNA methylation of 2002 CpG islands and 2771 gene promoters (-3.88-0.97kb), among which 1350 (67.43%) CpG islands and 2095 (75.60%) promoters contained region-specific DNA methylation. We observed significant and distinct functional enrichment in genes with specifically methylated promoters in each epididymal regions, but these DNA methylations did not show significant correlation with repressed gene transcription in the mature epididymis. Conclusively, we investigated the regional miRNA expression and DNA methylation in the rat epididymis and revealed a potential role of miR-200 family in gene expression regulation between caput and cauda. This may contribute to the distinct physiological function in sperm maturation / storage of caput / cauda epididymis.

## Introduction

Along the mammalian epididymal tubule, epithelial cells secrete proteins in a region-specific manner, creating an ever-changing luminal environment for sperm maturation and storage [1–4]. This feature is attributed to the uniquely fine-tuned spatial gene expression pattern of epididymal epithelial cells [5–6]. In mammalian epididymis, region-specific gene expression is crucial to sperm maturation, storage and male fertility, and it implicates stringent gene regulation mechanisms as well. Androgen is powerful in activating epididymal gene transcription through its receptor (AR), and hitherto a number of androgen-responsive genes and pathways have been identified [7–9]. Comparatively however, other potential mechanisms in this multi-level gene regulation network were far less investigated and understood.

In mammalian cells, DNA methylation and miRNAs modulate target gene activities without altering the DNA sequence and respectively regulate gene expression at transcriptional and post-transcriptional levels. DNA methylation is a biochemical process in which a methyl group is added to the cytosine in CpG dinucleotides to form 5-methylcytosine by methyltransferases [10]. CpG islands are regions with a high frequency of CpG sites, and DNA methylation on the CpG islands in the gene promoter region gives rise to the stable repression of the target genes [11].On the other hand, miRNAs are endogenous small RNAs that play important gene-regulatory roles by pairing to the 3’ UTR region of the target mRNAs to direct their post-transcriptional repression [12]. Mammalian miRNAs recognize their target mRNAs by 6–8 nucleotides known as the seed region [13]. miRNAs with sequence similarity in the seed regions often share analogous biological functions and can be clustered as miRNA families [14].

Gene expression regulation by DNA methylation and miRNAs has been reported in many contexts, including their physiological roles in the proper differentiation and development of various mammalian cells and tissues [15–18], and the pathological roles in complex diseases [19], especially cancers [20–21]. Nevertheless, not much is known about the involvement of DNA methylation and miRNAs in gene expression control in the epididymis. It was reported that the epididymal spatiotemporal expression of mouse Rhox5 gene was regulated by the differentially methylated promoter region that recruited androgen receptors as the transcription factor [22], indicating an important role of DNA methylation in transcription activation of epididymal genes. However, we still lack of a general picture of genome-wide DNA methylation patterns throughout the epididymis. For epididymal miRNAs, previous studies have set foot into their functions in epididymal postnatal development [23–28]. More than 100 ‘age-specific’ miRNAs were identified by microarray [23] and epididymal miRNAs were shown to be essential for the epithelium differentiation and sex steroid signaling through the study of the epididymal conditional Dicer1 knockout mouse model [24]. Additionally, specific miRNAs such as miR-200a [26], miR-200c [27], miR-335 [28] and miR-29a [25] were identified to regulate epididymal development by targeting β-catenin, E-cadherin, Zeb1, Nasp and etc. Comparatively, less attention has been paid on how miRNAs contribute to the maintenance of region-specific gene expression and intercellular communication in the mature epididymis, where miRNAs expressed by the epididymal epithelial cells can either function *in situ*, or be released into the luminal fluid via epididymosomes [29]. A previous study has identified the miR-888 cluster and miR-215 to be expressed in a region-specific pattern in the human epididymis [30],and further research is needed to discover more differentially expressed miRNAs in the epididymis and compare their conservativeness among species.

To this end, we investigated and portrayed the epididymal regional DNA methylation and miRNA expression profiles in rats by two microarray-based approaches: the Methyl-DNA immunoprecipitation-chip (MeDIP-chip) and miRNA microarrays. We sought to identify the differentially methylated gene promoters and expressed miRNAs. To determine whether and how epididymal region-specific promoter methylation and miRNA expression contribute to the mRNA expression, we further adapted the rat epididymal regional gene expression data from a previous study [31]. By ‘gene set enrichment analysis (GSEA)’ [32], a computational method widely used for association studies in many contexts [33–34], we performed correlation analyses of epididymal mRNA-miRNA and mRNA-DNA methylation in a genome-wide scale.

## Materials and Methods

### Sample preparation

Male 450g Sprague-Dawley rats were purchased from the Animal Center of the Chinese Academy of Sciences (Shanghai, China). They were kept in the animal housing at our institute before manipulation. Food and water were freely available throughout the experiments. All protocols in this study were conducted according to the approval of the Institute Animal Care Committee of Shanghai Institute of Biochemistry and Cell Biology (Permit Number: SYXK2007-0017).To prepare tissue samples, rats were sacrificed by CO_2_ inhalation. 10 epididymides of 5 rats were excised, freed of connective tissues and divided into 4 anatomical regions (initial segment (IS), caput (Cap), corpus (Cor) and cauda (Cau)). Tissues were finely minced and rinsed 3 times with cold PBS to remove epididymal luminal fluid and spermatozoa, and then frozen in liquid nitrogen for MeDIP and miRNA extraction.

### MeDIP-chip

Pooled biological samples instead of biological replicates were used in this study. The IS, Cap, Cor and Cau segments of all the rats were respectively pooled together for genomic DNA extraction with TIANamp Genomic DNA Kit (TianGen) according to the manufacture’s protocol. Genomic DNA was dissolved in DNase-free water and the concentration was determined by Nanodrop2000c (Thermo). Genomic DNA samples were sonicated to generate fragments with the average size of 400bp. Briefly, 20μg of genomic DNA was diluted in 300–450μl IP buffer (10mM sodium phosphate buffer (pH = 7.0), 140mM NaCl, 0.05% Triton X-100) in a 1.5ml eppendorf tube. The sonicator was turned on 30s and then off 30s for 20 times. After sonication, 4μl of the sample was loaded on a 2% agarose gel to verify fragment size of DNA. Then each eppendorf tube containing 6–7 μg sonicated DNA was incubated for 10 min in boiling water, and immediately cooled on ice for 10 min for heat-denature. 5μg anti-5’-methylcytosine antibody was used to immunoprecipitate methylated DNA fragments in IP buffer with Dynabeads M-280 sheep anti-mouse IgG beads (Invitrogen). After 2h incubation at 4°C, the beads were collected and resuspended in IP buffer plus proteinase K and further incubation was performed at 50°C for 3h to digest proteins. The recollected beads were discarded and the supernatant was transferred to a fresh tube for purification. The concentration of resulting DNA fragments was determined by Nanodrop2000c (Thermo).

DNA methylation profiles of4 epididymal regions (IS, Cap, Cor and Cau) were generated by the NimbleGen Multiplex RN34 CpG Promoter MeDIP-chip platform at KangChen. All procedures were carried out according to the manufacturer’s protocol. This array contains probes for 14723 gene promoters covering -3.88kb upstream to 0.97kb downstream and 15809 CpG islands. The ACME (Algorithm for Capturing Microarray Enrichment) algorithm [35] supplied by NimbleScan was employed for peak-finding. The ACME algorithm stipulates that a fixed-length window is slid along the whole length of each chromosome, testing at each probe whether the surrounding window is enriched for high-intensity probes relative to the rest of the array using a one-sided Kolmogorov-Smirnov (KS) test. Each probe has a corresponding p-value score (Peak score: the average-log10 (P-value) from probes within the peak) and a threshold is set to select regions that are methylated in the test sample. Here, the following parameters were used for finding significant peaks: sliding window with— 750bp; mini probes—2; Peak score minimum cutoff—2; maximum spacing between nearby probes within peak— 500bp. (The MeDIP-chip procedure was shown in S1 Fig and results are available in S1 Table)

### miRNA microarrays

Pooled biological samples instead of biological replicates were used in this study. The IS, Cap, Cor and Cau segments of all the rats were respectively pooled together for small RNA extraction with miRcute miRNA isolation kit (TianGen) according to the manufacture’s protocol. miRNAs were dissolved in DEPC treated water and the concentration was determined by Nanodrop2000c (Thermo).

miRNA microarrays were performed as previously described [25]. Briefly, RNA samples were isolated, size fractionated, and labeled with Cy3 or Cy5. Samples were hybridized to dual-channel microarray using the μParaflo microfluidics chips of LC Sciences (Houston, TX). This array contained probes for rat, mouse and human miRNAs listed in Sanger miRBase Release 11.0. The reverse transcription, cRNA synthesis, labeling and hybridization with Affymetrix GeneChip Rat Genome 230 2.0 Array were conducted following the standard Affymetrix protocol. The background-subtracted data were normalized by the LOWESS and quantile normalization methods. The log2-fold-change values were calculated for each miRNA by comparing their expression between any two epididymal regions. Meanwhile, statistical significance of fold change (P value) was inspected through the Student’s *t* test. Volcano plot was used to portray the miRNA expression difference between two epididymal regions, in which the x axis indicated the log2 fold change value and y axis indicated the negative log10 P value. (miRNA microarray results are available in S3 Table)

### mRNA expression data extraction

The rat epididymal regional mRNA expression data were extracted from a published study [31]. In that study, the rat epididymides were divided into 19 anatomical regions and respectively profiled for mRNA expression. As the 19 regions were the subdivision of IS (region 1–4), Cap (regions 5–9), Cor (regions 10–13) and Cau (regions 14–19), we combined the mRNA expression data of corresponding regions into IS, Cap, Cor and Cau by taking the average of each region. The extracted mRNA expression data were used for expression correlation and GSEA studies.

### qPCR verification of miRNA expression

A reverse transcription step was performed prior to quantitative PCR (qPCR). Briefly, a stem-loop RT primer was hybridized to the miRNA molecule and then reverse transcribed in a pulsed RT reaction. 1 μg of total RNA extracts were denatured and mixed with 62.5 μM of each dNTP, 50 nM of the stem-loop primer, and 1 unit of M-MLV reverse transcriptase (Promega) at 16°C for 30 min, 42°C for 1h, 16°C for 10 min, and then incubated on ice.

qPCR was performed by using the SYBR Green (TOYOBO) assay with Rotor-Gene 3000 (Corbett). Briefly, for each miRNA, SYBR Green I Master Mix was mixed with 0.5 μM of a specific forward primer and a universal reverse primer and 5 μl of the RT template for a total volume of 20 μl. Samples were incubated at 95°C for 1 min, followed by 40 cycles of 95°C for 10 s, 60°C for 15 s and 72°C for 20 s. Data were normalized to U6 snRNA. Primers used were listed in Table 1.

**Table 1:** List of primers used in this study.

**Primers for RT**	**Sequences (5' to 3')**	
rno-let-7a	GTCGTATCCAGTGCAGGGTCCGAGGTATTCGCACTGGATACGACAACTAT	
rno-miR-200a	GTCGTATCCAGTGCAGGGTCCGAGGTATTCGCACTGGATACGACACATCG	
rno-miR-200b	GTCGTATCCAGTGCAGGGTCCGAGGTATTCGCACTGGATACGACGTCATC	
rno-miR-200c	GTCGTATCCAGTGCAGGGTCCGAGGTATTCGCACTGGATACGACCATCAT	
rno-miR-141	GTCGTATCCAGTGCAGGGTCCGAGGTATTCGCACTGGATACGACCCATCT	
rno-miR-429	GTCGTATCCAGTGCAGGGTCCGAGGTATTCGCACTGGATACGACACGGCA	
rno-miR-664	GTCGTATCCAGTGCAGGGTCCGAGGTATTCGCACTGGATACGACTAGGCT	
rno-miR-327	GTCGTATCCAGTGCAGGGTCCGAGGTATTCGCACTGGATACGACACCCTC	
**Primers for qPCR**	**Sequences (5' to 3')**	
rno-let-7a Forward	AAGTTGCATGAGGTAGTAGGTTG	
rno-miR-200a Forward	ACGGCACTAACACTGTCTGG	
rno-miR-200b Forward	AATCCGGATAATACTGCCTGGT	
rno-miR-200c Forward	AAGCGGATAATACTGCCGGG	
rno-miR-141 Forward	ACGGCACTAACACTGTCTGG	
rno-miR-429 Forward	AAGCGACCTAATACTGTCTGGT	
rno-miR-664 Forward	CGCGGCCTATTCATTTACTCC	
rno-miR-327 Forward	ACAGCTCCTTGAGGGGCAT	
Universal Reverse	CAGTGCAGGGTCCGAGGT	
rat U6 snRNA Forward	GCTTCGGCAGCACATATACTAA	
rat U6 snRNA Reverse	CGAATTTGCGTGTCATCCTT	
**Primers for Molecular Cloning**	**Forward (5' to 3')**	**Reverse (5' to 3')**
rRab21 3’ UTR	CGCTCGAGCGGCTCACGCCTGCGAACTAAACAA	CGGCGGCCGCGCACCTTGCGCTTGTAAAAGCAGAT
rBcap29 3’ UTR	CGCTCGAGAGTAACTTTACAAAAGAAGATTGTG	CGGCGGCCGCTACATTTTCTTTTATATGTATGCAT
rDek 3’ UTR	CGCTCGAGGATAGAGGACAGATGGCTTGTTCTC	CGGCGGCCGCAAATACAAGAAACTTAAAACATTGA
rSlc23a1 3’ UTR	CGCTCGAGAACTACCTCTATGAAAGGAGGCACG	CGGCGGCCGCAGTCTGAAAGTATAGATTTATTTTC

### Prediction of miRNA targets

To increase the prediction accuracy, putative targets of miRNAs were predicted by miRWalk, an integrative prediction method containing Diana-microT, RNA22, miRanda, miRDB, TargetScan, RNAhybrid, PITA, and PICTAR algorithms [36]. In practice, genes predicted by over 5 algorithms in the miRWalk were considered as targets of a certain miRNA.

### GSEA analysis

GSEA is a computational method for association studies by examining whether the expression profile of a certain gene set have a significant tendency between two concerned biological states [32] (in this case, Cap and Cau epididymal regions). First, a predefined rank gene list was made by sorting all genes according to their expression levels between these two epididymal regions. The extracted mRNA expression profiles of Cap and Cau [31] were utilized to build the predefined rank gene list. Then, a certain set of genes were ‘mapped’ to the predefined rank gene list and GSEA calculated the enrichment score, P value and false discovery rate (FDR) of this gene set to see whether these genes were significantly enriched in the top or bottom of the predefined rank gene list [32]. The enrichment would be considered ‘significant’ when the P value<0.05 with FDR<20%. In our study, we used GSEA to test the following gene sets: 1. genes with different methylation status (i.e. genes with Cap / Cau specific methylated promoters) and 2. target genes of differentially expressed miRNAs between Cap and Cau epididymis. In practice, the analysis was performed using the GSEA software (version 2.0.14) [32], where the parameter of “Enrichment statistic”was set to the “classic” item, and the parameter of “Metric for ranking genes” was respectively set to “Signal2Noise”, “tTest”, “Ratio_of_Classes”, “Diff_of_Classes” and “log2_Ratio_of_Classes”. 1000 permutations were carried out to evaluate FDR and P value of the enrichment score with permutation type set to the “gene_set”.

### Luciferase reporter constructs and assay

Luciferase reporter assay was performed to verify the *bona fide* regulation of miR-200 family on 4 putative target genes (Bcap29, Rab21, Slc23a1 and Dek) whose 3’ UTR regions contained potential target sites of the 5 miRNAs, in whole or in part. Briefly, the 3’ UTR region of each gene was respectively amplified (The primers used for amplification were listed in Table 1). After being cut with XhoI and NotI, the amplified products were subcloned into the downstream of the *Renilla* luciferase gene of the psiCHECK-2 vector (Promega). HEK293T and Hela cells were seeded in 24-well plates 24 h before transfection. Cells were transiently transfected with 100 ng of each of the above 4 constructs and together with respectively 40 pmol mimics of miR-200a, miR-200b, miR-200c, miR-141, miR-429 and miR-NC (Negative control miRNA, 5’- UUCUCCGAACGUGUCACGUTT -3’). Lipofectamine 2000 reagent was used for co-transfection of plasmids and RNA oligonucleotides. The luciferase activities were measured with the Dual Luciferase Assay kit (Promega) by Synergy NEO (BioTek) 24 h after transfection. *Renilla* luciferase activity was normalized to *Firefly* luciferase activity.

### Gene functional analysis

To further explore the potential functions of epididymal promoter methylated genes, we performed Gene Ontology (GO) enrichment analysis by the Functional Annotation Tools of DAVID (http://david.abcc.ncifcrf.gov/) [37] and iGepros (http://www.biosino.org/iGepros/) [38]. In practice, three main categories of GO (i.e. cellular component (CC), biological process (BP), and molecular functions (MF)) were separately analyzed with a threshold of P value<0.05.

## Results

### Profiling DNA methylation along the rat epididymis

**Fig 1:**
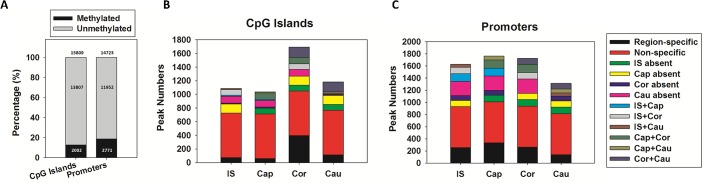
DNA methylation status of gene promoters and CpG islands among 4 regions of rat epididymis. (A) Percentage of DNA methylation peaks. (B) Regional distribution of CpG island methylation peaks. (C) Regional distribution of promoter methylation peaks.

To capture the distinct features of regional DNA methylation status along the rat epididymis, the MeDIP-chip approach was implemented to detect methylated cytosines from 14723 gene promoters and 15809 CpG islands in 4 regions of the epididymal tubule. Among them, 2771 (18.82%) promoters and 2002 (12.66%) CpG islands contain DNA methylation peaks in the epididymis (Fig 1A and S1 Table). To verify the MeDIP-chip results, we examined the promoter methylation status of Mbp, Uox, Slc7a1, Cdkl1 and Cdx2 by bisulfite sequencing PCR (BSP) because they had different ‘Peak scores’ according to the MeDIP-chip results (S1 Table and S1 Fig), i.e. Mbp had the highest peak score among all genes; Uox, Slc7a1 and Cdkl1 were randomly picked genes with lower peak scores (still above threshold, indicating ‘methylated’); Cdx2 was a randomly picked gene with a peak score<2 (indicating ‘unmethylated’). By BSP validation, we found that although the ‘Peak score’ of each gene didn’t correlate with their actual promoter methylation levels, the MeDIP-chip data of the tested genes all agreed with the BSP results (S1 File and S1 Fig).

The regional distribution of methylation peaks was summarized in Table 2. 32.57% (652 in 2002) CpG islands and 24.40% (676 in 2771) promoter DNA methylation peaks were detected in all segments of the epididymis with no region-specificity. The remaining region-specific DNA methylation peaks were localized in 1350 (67.43%) CpG islands and 2095 (75.60%) promoters and distributed in 1, 2 or 3 of the 4 regions (Fig 1B and 1C). Among all 4 regions, cauda epididymis has the fewest promoter methylation peaks, suggesting minimum transcription repression (Fig 1C).

**Table 2:** Summary of DNA methylation peak distribution.

	CpG Islands			Promoters		
	Quantity	Percentage		Quantity	Percentage	
**Examined**	15809	100%		14723	100%	
**Methylated in …**						
**at least 1 region**	2002	12.66%	100%	2771	18.82%	100%
**IS**	1088	6.88%	54.34%	1630	11.07%	58.82%
**Caput**	1036	6.55%	51.75%	1765	11.99%	63.71%
**Corpus**	1690	10.69%	84.42%	1725	11.72%	62.25%
**Cauda**	1182	7.48%	59.04%	1314	8.92%	47.42%
**All 4 regions**	652	4.12%	32.57%	676	4.59%	24.40%
**Only in IS**	72	0.46%	3.60%	254	1.73%	9.17%
**Only in Caput**	60	0.38%	3.00%	334	2.27%	12.05%
**Only in Corpus**	397	2.51%	19.83%	262	1.78%	9.46%
**Only in Cauda**	115	0.73%	5.74%	137	0.93%	4.94%

### Functional enrichment of epididymal genes with promoter DNA methylation

We performed GO enrichment analysis to further explore the function of epididymal promoter methylated genes. Detailed information of enrichment analysis was shown in S2 Table. First, genes with methylated promoters in all 4 epididymal regions have significantly enriched functions in the categories of “ion transport”(0.76%, P = 8.83E-6), “sexual reproduction”(0.47%, P = 1.11E-4), “spermatogenesis”(0.36%, P = 2.14E-4) and “meiosis”(0.15%, P = 0.0010). Additionally, these genes were significantly enriched in the cellular components of “plasma membrane”(2.34%, P = 2.66E-9) and “intermediate filament”(0.27%, P = 4.75E-6). Then, we examined the function of genes with region-specific promoter methylation. IS specific methylated genes enriched in function categories of “cell death”(0.75%, P = 0.0018), “response to cytokine stimulus”(0.32%, P = 0.0124) and “apoptosis”(0.59%, P = 0.0157), and gene products significantly enriched in “integral to membrane”(4.40%, P = 0.0019). Caput specific methylated genes enriched in function categories of “regulation of cellular localization”(0.56%, P = 4.54E-4), “regulation of secretion”(0.52%, P = 4.57E-4), “regulation of cytokine production”(0.37%, P = 0.0018) and “response to hormone stimulus”(0.75%, P = 0.0020), and gene products significantly enriched in the “extracellular region”(1.50%, P = 0.0024) and “cell surface”(0.52%, P = 0.0144). Corpus specific methylated genes enriched in function categories of “reverse cholesterol transport”(0.14%, P = 0.0081), “spermatogenesis”(0.42%, P = 0.0136) and “response to inorganic substance”(0.42%, P = 0.0206), and gene products significantly enriched in “intracellular non-membrane-bounded organelle”(1.92%, P = 0.0040) and “cytoskeleton”(1.08%, P = 0.0176). Cauda specific methylated gene enriched in function categories of “cell proliferation”(0.97%, P = 3.45E-5), “positive regulation of immune response”(0.48%, P = 0.0130) and “lymphocyte activation”(0.48%, P = 0.0232), and gene products significantly enriched in “axon”(0.68%, P = 0.0028) and “cell fraction” (1.46%, P = 0.0079). Our data indicated that genes with specific promoter methylation in each epididymal region show significantly distinct function enrichment and cell localization.

### Profiling miRNA expression along the rat epididymis

**Fig 2:**
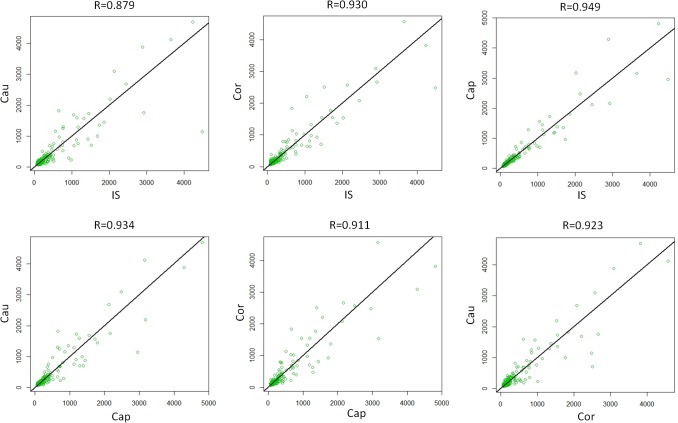
Regional difference of miRNA expression in rat epididymis. miRNA expression values (microarray signals) between 2 regions were displayed by scatter plot. Pearson correlation coefficient was used to characterize the difference.

Using miRNA microarray we revealed the expression level of 278 miRNAs along the rat epididymis (S3 Table). We calculated the Pearson correlation coefficient of the miRNA expression between each two regions to determine the degree of similarities in post-transcriptional regulation and function. The correlation value ranged from 0.879 (IS vs Cau) to 0.949 (IS vs Cap) (Fig 2). Using the same method, we calculated the rat epididymal regional mRNA expression correlation of the extracted data [31]. The mRNA correlation value was between 0.726 (IS vs Cau) to 0.916 (Cor vs Cau) (S2 Fig). Adjoining epididymal regions show higher correlations than separate ones in both mRNA and miRNA profiles, which implied the gradual change of mRNA / miRNA expression and epididymal function. Furthermore, we observed that unlike a number of mRNAs which showed ‘on-off’ expression pattern between two epididymal regions (S2 Fig) (for example Cst8, Lcn5 and Defb42 [31]), the miRNA expression was more consistent among different regions of the epididymis (Fig 2).

### miR-200 family was more expressed in the caput than in the cauda epididymis

**Fig 3:**
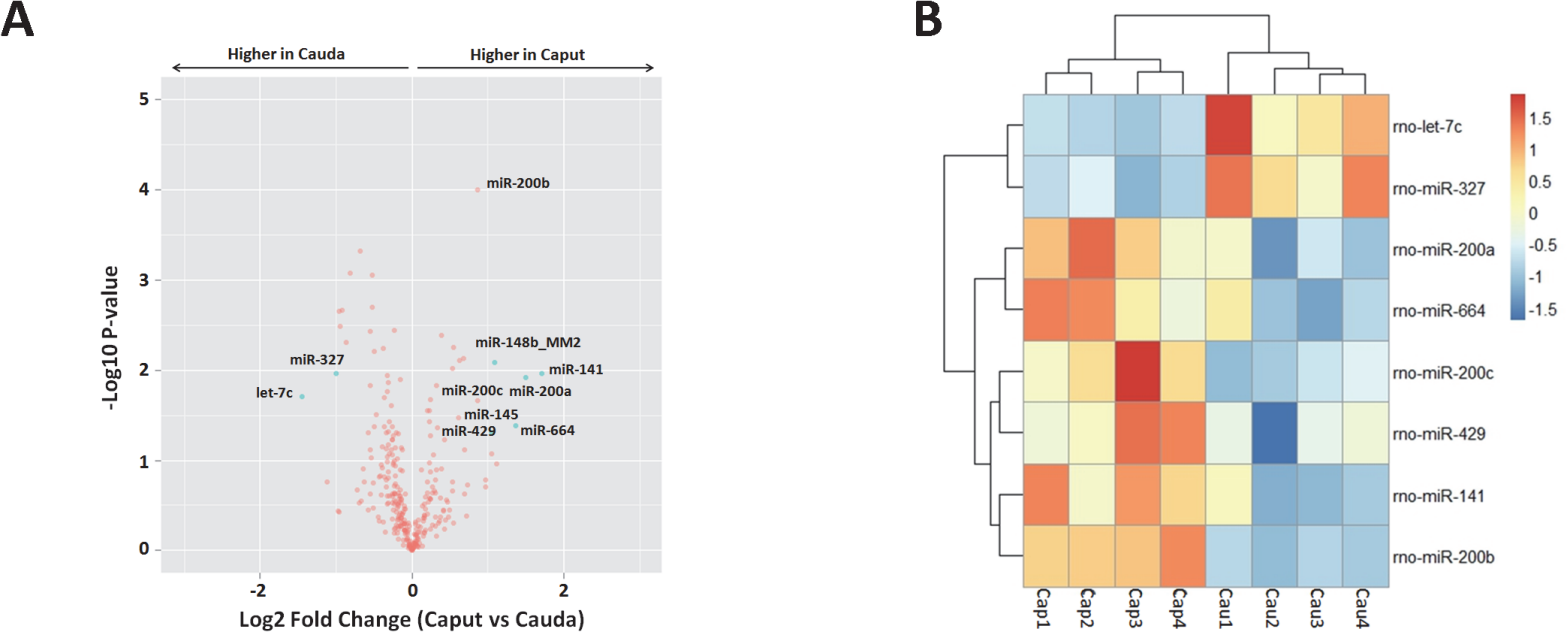
miRNA expression difference between caput and cauda epididymis. (A) miRNA expression differences and P values of caput and cauda epididymis were displayed by volcano plot. Blue dots indicated miRNAs with Log2 fold change>1 and P value<0.05. (B) Hierarchical clustering of differentially expressed miRNAs (Log2 fold change>1, P value<0.05) and miR-200 family between caput and cauda epididymis. Cap1-4 and Cau1-4 means repetition.

We further sought to compare the miRNA expression level between caput and cauda, the two epididymal regions that have distinct biological functions in sperm maturation and storage. The diversity in miRNA expression of caput and cauda epididymis was characterized by volcano plot, which presented both the fold change of expression and P values (Fig 3A).

7 miRNAs that met the criteria ofLog2 fold change>1 and P value<0.05 were represented in blue dots and shown in Table 3. Among them, miR-664, miR-141, miR-145, miR-148b_MM2, miR-200a showed higher expression in the caput while miR-327 and let-7c in the cauda. As two members of the miR-200 family (miR-200a and 141) were observed, we further examined the expression pattern of the remaining three miRNAs (miR-200b, 200c and 429). Expression of both miR-200b and miR-200c were significantly higher in the caput (P<0.05) with around 2 fold in change and miR-429 was also higher in the caput, with P = 0.060 and fold change of 1.35. The caput / cauda differentially expressed miRNAs were clustered and displayed by heatmap (Fig 3B).

**Table 3:** Differentially expressed miRNAs between caput and cauda epididymis.

miRNA_name	Caput	Cauda	fold_change	log2_fold_change	P value
**rno-miR-141**	735.56	224.03	3.28	1.72	0.0108
**rno-miR-200a**	832.25	293.44	2.84	1.50	0.0120
**rno-miR-664**	2948.44	1141.00	2.58	1.37	0.0418
**rno-miR-148b_MM2**	245.75	115.25	2.13	1.09	0.0083
**rno-miR-145**	238.60	115.66	2.06	1.04	0.0488
**rno-miR-327**	648.75	1304.56	0.50	-1.01	0.0110
**rno-let-7c**	665.81	1820.56	0.37	-1.45	0.0197

### qPCR verification of miRNA expression

**Fig 4:**
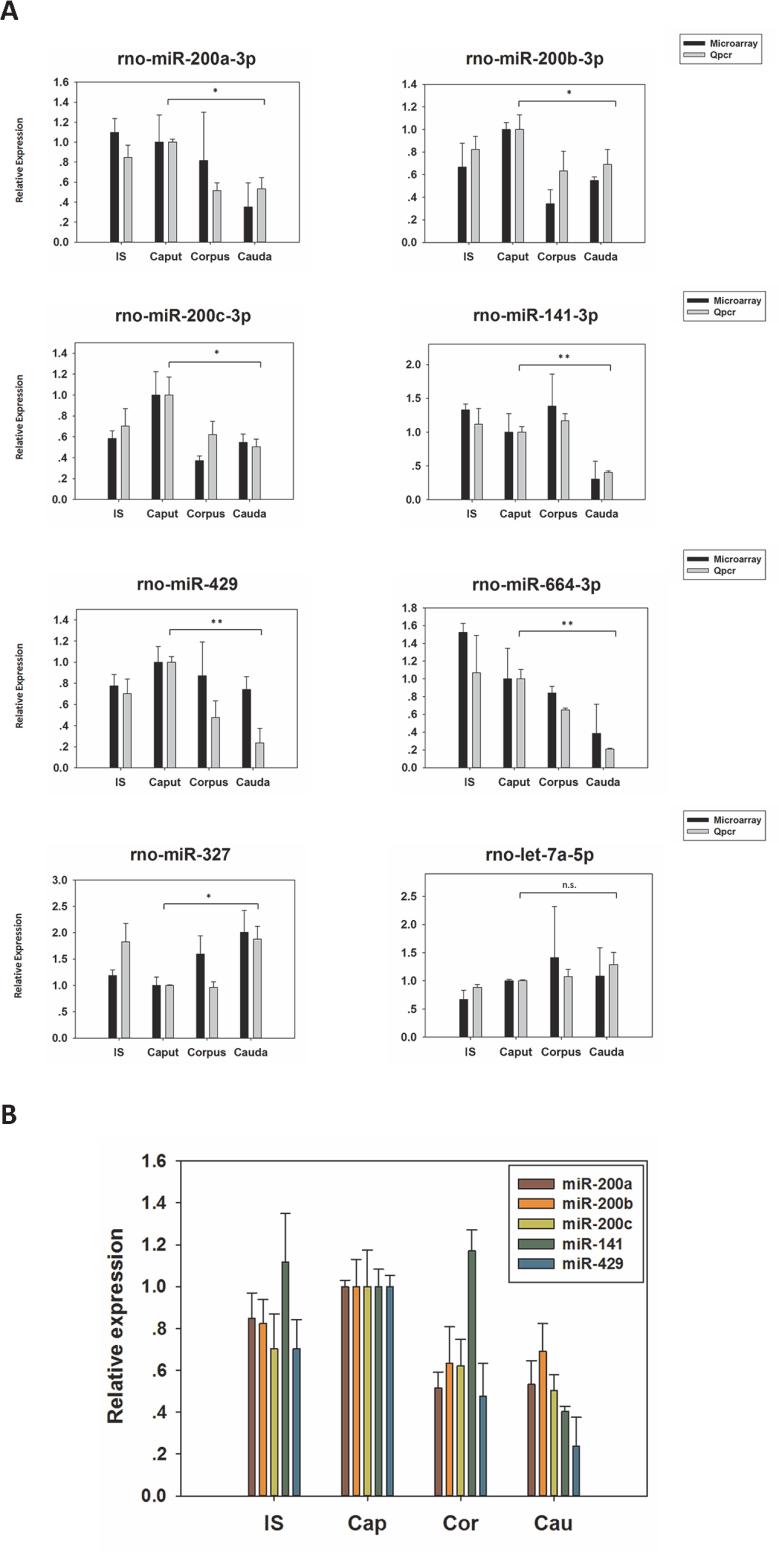
qPCR verification of epididymal regional miRNA expression. (A) The expression of miR-200a, miR-200b, miR-200c, miR-141, miR-429, miR-664, miR-327 and let-7a was examined from the IS, Cap, Cor and Cau epididymis of 5 SD rats by qPCR and compared with microarray results. The values of Cap were normalized to 1 in each group. (B) The regional expression of miR-200 family in rat epididymis examined by qPCR. Data are means ± SEM from 3 independent experiments. *: P≤0.05, **: P≤0.01.

Epididymal regional expression of 8 miRNAs was verified by qPCR (Fig 4A). Among them, 5 miR-200 family miRNAs, together with miR-664 and miR-327, were shown by microarray to be differentially expressed between caput and cauda epididymis. The expression of let-7a was non-region-specific. Both microarray and qPCR data were normalized to caput expression and regional trend of expression for each miRNA was compared.

The qPCR data of all the 8 miRNAs confirmed the microarray result by showing same trend. Therein, significant expression variations were revealed by qPCR in 7 miRNAs between caput and cauda, with more conspicuous difference in miR-429 and miR-664 (Fig 4A). Taken together, cauda expression of the miR-200 family were 30%- 70% lower than the caput level (Fig 4B), implicating potential roles of this miRNA family in maintaining region-specific gene expression between the two regions.

### GSEA analysis revealed negative expression correlation between miR-200 family and their target genes

**Fig 5:**
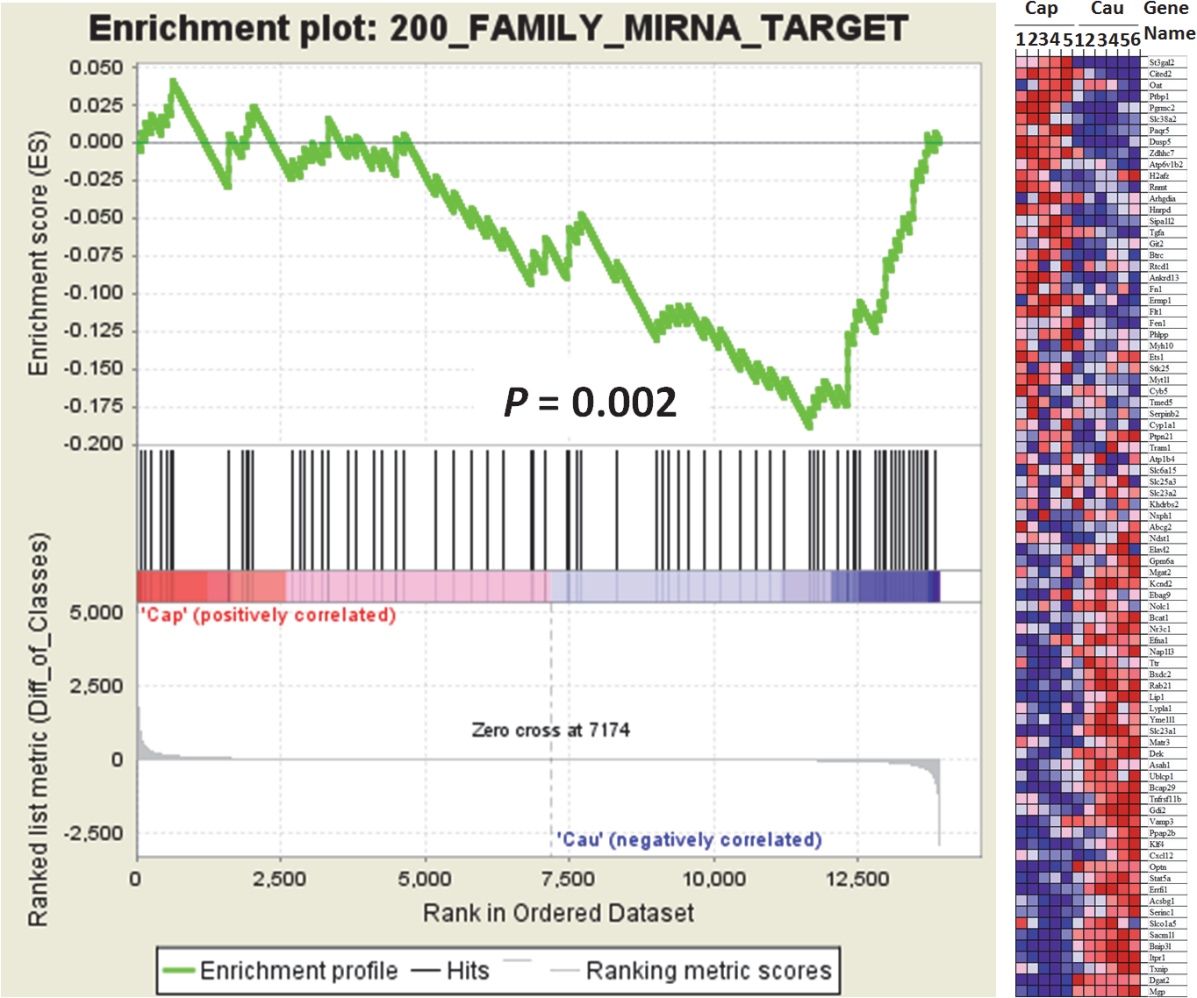
Results of GSEA analysis for target genes of miR-200 family. Left panel: enrichment score of putative miR-200 target genes against their expression profile of caput and cauda epididymis. The x axis was the Cap/Cau level, and the y axis was the enrichment score of these genes. Right panel: heat map of miR-200 target gene expression in caput and cauda region (Cap1-5 and Cau1-6 reflected the subdivision of each region in the study performed by Jelinsky et al., [31]).

To study how miR-200 family contributed to epididymal region-specific gene expression, we performed GSEA analysis to examine whether putative target genes of miR-200 family were coordinately more or less expressed between caput and cauda epididymis. For the GSEA analysis, a predefined ranked gene list was first generated by comparing the expression level of each gene in the caput and cauda region(i.e. the higher Cap/Cau level, the higher position a gene was located in the ranked list; the lower Cap/Cau level, the lower position it was located). Then the set of putative miR-200 target genes was investigated whether they were significantly sat in the top or bottom of the ranked list. As shown in Fig 5 and Table 4, for the miR-200 targets, their enrichment score achieved -2.01 with a significant P value (0.002) and FDR (0.9%, much less than 20%) when the parameter of “metric of ranking genes” was “diff_of_Classes” and the parameter of “Enrichment statistic” was “classic”. This result indicated that the putative miR-200 family target genes were significantly located in the bottom of the gene list and their expression in the cauda was statistically higher than in the caput. Given the higher expression of miR-200 family in the caput region, it was suggested that differentially expressed miR-200 family down-regulated their target genes in the epididymis. To study whether other region-specific miRNAs and promoter methylations potentially regulated epididymal gene expression, we performed GSEA analysis on other gene sets (i.e. 1. target genes of caput more expressed miRNAs; 2. target genes of cauda more expressed miRNAs; 3. genes with caput specifically methylated promoters; 4. genes with cauda specifically methylated promoters), but we didn’t observe significant enrichment of these gene sets (S3 Fig and S4 Table).

**Table 4:** Result of GSEA analysis for miRNA-200 family target genes.

metric for ranking genes	parameter p = 0		
	ES	P value	FDR
**Signal2Noise**	-1.470	0.069	0.263
**tTest**	-1.460	0.085	0.245
**Ratio_of_Classes**	-1.330	0.127	0.219
**diff_of_Classes**	-2.010	0.002	0.009
**log2_Ratio_of_Classes**	-1.320	0.169	0.233

Note: ES stands for enrichment score, and FDR stands for false discovery rate.

### *Bona fide* regulation of miR-200 family on 4 putative target genes

**Fig 6:**
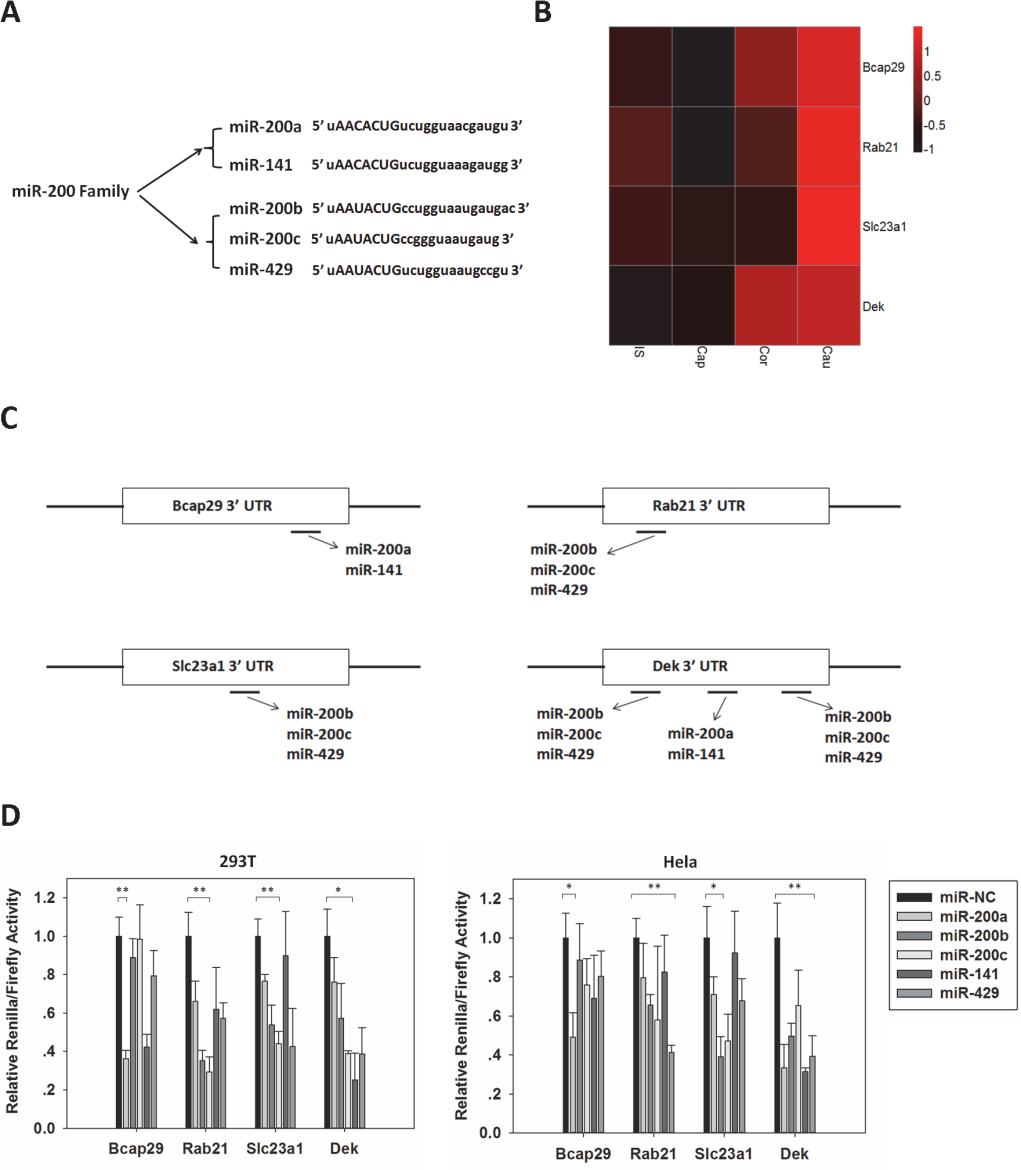
Luciferase reporter assay of miR-200 family action on Bcap29, Rab21, Slc23a1 and Dek 3’ UTR. (A) Two subfamilies of the miR-200 family with slightly different seed regions. (B) Epididymal regional expression of the 4 genes. (C) Predicted targeting of miR-200 family on target genes. (D) Luciferase reporter assay performed on HEK293T and Hela cell lines. Data are means ± SEM from 3 independent experiments. *: P≤0.05, **: P≤0.01.

The miR-200 family comprises of 5 miRNAs: miR-200a, miR-200b, miR-200c, miR-141 and miR-429. These miRNAs can be further divided into 2 subgroups based on their slightly different seed region sequences (Fig 6A), and the 2 subgroups are respectively clustered at 2 locations in the genome [39]. Dual luciferase reporter assay was performed to confirm the targeting and regulation of miR-200 family on 4 predicted target genes: Bcap29, Rab21, Slc23a1 and Dek. All the 4 genes were more expressed in the cauda than caput (Fig 6B) based on the epididymal mRNA microarray data [31], and their 3’ UTR regions contained potential target sites of the miR-200 family, in whole or in part (Fig 6C). In detail, Bcap29 was predicted to be targeted by miR-200a and 141, Rab21 and Slc23a1 by miR-200b, 200c and 429, and Dek by all the 5 miRNAs. In addition, no promoter of these 4 genes was methylated in the epididymis based on our data and none of them was shown to respond to androgen [8,40], which excluded the possible influence of these two mechanisms on their expression. 24h after co-transfection of psiCHECK-2 vector containing 3’ UTR region of each gene and the corresponding synthetic mimic miRNAs or miR-NC, the *Renilla* and *Firefly* luciferase activities were measured. Compared to the miR-NC group, mimic miRNAs significantly diminished the relative *Renilla/Firefly* activity of their predicted target genes in both 293T and Hela cells (Fig 6D). This result confirmed the *bona fide* interaction and regulation of miR-200 family to their targets.

### Functional enrichment of miR-200 family target genes

We performed GO enrichment analysis to further explore the potential functions of miR-200 family target genes. Detailed information of enrichment analysis was shown in S5 Table. Functions of miR-200 family target genes were mainly enriched in the categories of “anti-apoptosis”(8.04%, P = 0.0002) (Ets1, Stat5a, Bnip3l, Tgfa, Nr3c1, Fn1, Cited2), “carboxylic acid transport”(6.90%, P = 0.0019) (Slc23a1, Slc38a2, Slc23a2, Serinc1, Slc6a15, Slco1a5), “transmembrane receptor protein tyrosine kinase signaling pathway”(6.90%, P = 0.0063) (Txnip, Flt1, Ndst1, Efna1, Stat5a, Tgfa) and “blood vessel development”(6.90%, P = 0.0126) (Flt1, Efna1, Tgfa, Ppap2b, Cxcl12, Cited2), suggesting that miR-200 family contributed to these functional diversities between caput and cauda epididymis. More detailed and specific actions of miR-200 family on epididymal development, sperm maturation and storage are needed to be explored in depth in the future studies.

## Discussion

The epididymis plays a pivotal role in mediating post-testicular sperm maturation, storage and acquisition of fertility. The highly regionalized epididymal tubule featured with distinct luminal microenvironments enables the multi-step maturation and final storage of spermatozoa that pass by. Over the past decade, epididymal region-specific gene expression has been proved to be responsible for the establishment and maintenance of luminal microenvironments, and the androgen—AR has been shown to be a powerful epididymal gene transcription activator. However lots of questions still remain unsolved. One major puzzle is why many non-androgen responsive genes also express in region-specific patterns in the epididymis?

Gene regulation system is a rigorous and multi-level network that creates, supervises and maintains the steady gene expression pattern. We hypothesized that other control mechanisms also play essential and indispensable parts in the epididymis. Therein, DNA methylation and miRNAs were known to play extensive and powerful roles in gene expression regulation at transcriptional and post-transcriptional levels. The microarray technology provides an efficient and cost-effective method for genome-wide profiling with high sensitivity [41–42], and therefore we examined and portrayed the regional DNA methylation and miRNA expression patterns in the rat epididymis. The data generated in our study could serve as useful resources for future researches.

Similar to the epididymal regional difference of mRNA expression over the IS, caput, corpus and cauda regions, our data showed the region-specific patterns of both miRNA expression and DNA methylation, which implied a potential relevance between these two mechanisms and the epididymal transcriptome. Interestingly, miRNAs were not so differentially expressed as mRNAs. Since miRNAs in eukaryotic cells direct translational repression, mRNA destabilization, or a combination of the two effects [12], it is possible that differentially expressed upstream miRNAs affect downstream gene expression by degrading target mRNAs and give rise to the more specific regional mRNA pattern. Among epididymal differentially expressed miRNAs, the whole miR-200 family was more expressed in the caput, compared with cauda, which inferred a role of this miRNA family in the maintenance of epididymal regional gene expression between the two physiologically distinct regions. We then studied and confirmed the negative expression correlation and *bona fide* regulation of miR-200 family on their target genes between caput and cauda.

As an important miRNA family, miR-200 miRNAs were proved to have marked biological significance in many aspects, especially in the inhibition of epithelial-mesenchymal transition (EMT) and repression of metastasis and progression of different cancer types such as ovarian and mammary cancer [39,43–46]. Interestingly, in these studies, the miR-200 family members tended to simultaneously up- or down-regulated in response to a certain change of physiological state [39,46]. Similarly, in our study, all 5 miR-200 family miRNAs showed higher expression in the caput (Fig 4B). Based on these observations, the whole miR-200 family seemed to work as a group, which might achieve amplified biological effects. Moreover, as a powerful inhibitor of EMT and cancer metastasis and progression, whether the epididymal miR-200 family contributes to the rarity of epididymal cancer is an intriguing question that requires further investigation.

Previous studies reported the epididymal temporal expression of miR-29and miR-200 families [25–27]. The expression level of both miRNA families elevated along with postnatal development of the epididymis, suggesting that they exerted more significant functions in the mature epididymis. Comparatively, the miR-29 family showed consistent regional expression [25] while the miR-200 family were differentially expressed. The discrepancy of spatial expression pattern of the two miRNA families implied a potential diversity of their functions in the epididymis. The temporal and spatial manners of miR-200 family expression may contribute to epididymal development, regionalization and maintenance of function, as revealed by GO analysis that their target genes enriched in functions of anti-apoptosis, cell transportation and development. More specifically, channel proteins such as Slc23a1, Slc38a2, Slc23a2, Slc6a15 and Slco1a5 are under the regulation of miR-200 family. Among them, Slc23a1 was proved to be involved in the reabsorption in the renal tubule [47] and might have the same function in the epididymal tubule. The versatile small GTPase Rab21 was proved to not only function in the early endosome-mediated endocytic pathway [48], but also regulate cell adhesion and control endosomal traffic of beta1-integrins [49]. Therefore Rab21 might help maintain the epididymal integrin-mediated cell adhesion and motility. In this study, we verified Slc23a1, Rab21, Bcap29 and Dek as the *bona fide* targets of the miR-200 family. Other potential target genes and their functions in the epididymis are also worth probing in the future.

Promoter DNA methylation is powerful in silencing gene expression in the context of embryo development, differentiation and cancer, where the methylation of promoter DNAs negatively correlated with the gene expression level [15–16,50–51]. In the mouse epididymis, a previous study revealed that promoter methylation of Rhox5 repressed the AR binding and transcription activation, in both temporal and regional manners [22], suggesting the involvement of epididymal DNA methylation in gene expression control at the transcription level. In our study, we detected specific promoter methylation sites as well as ubiquitous methylation sites in 4 rat epididymal regions (Fig 1), but we didn’t observe epididymal DNA methylation in rat Rhox5 (S1 Table). Based on the differentially methylated state of Rhox5 promoter between mouse and rat, more analyses are needed to evaluate the whole epididymal DNA methylation between different species. By GO analysis, we found that many genes with statistically significant functional enrichments tended to have methylated promoters. For example, promoters of 45 ion transportation-related (Kcnj16, Slc5a3, Steap3, Plcz1, Camk2g and etc.), 28 sexual reproduction-related (Acr, Fkbp6, Ccin, Capza3, Dedd, Adad1 and etc.) and 21 spermatogenesis-related genes (Mak, Mael, Acsbg2, Spo11, Sohlh2, Krt9 and etc.) were methylated throughout the epididymis. In the caput, DNA methylation occurred on promoters of 40 genes whose corresponding proteins were localized in the extracellular region (Ccl4, Il10, Il1a, Il21, Fgf17 and etc.). In the cauda, 10 cell proliferation-related genes were methylated (Calca, Prok2, Tyr, Uhrf2, Crip3 and etc.). GO enriched functions of these specific promoter methylated genes did relate to the biological functions of each epididymal region to a certain extent, but their expression between caput and cauda didn’t have significant negative correlation as revealed by GSEA analysis. Similarly, among those epididymal ubiquitously methylated genes, some of them show repressed expression in the epididymis, such as Kcnj16, Slc5a3 and etc, while some others (Steap3, Camk2g, Atp2b4, etc) still have certain levels of expression [31]. These discrepancies suggested that the epididymal region-specific promoter DNA methylation pattern may not be a key determinant in maintaining region-specific gene expression pattern in the mature epididymis. But why and how this pattern was established in the first place, especially on genes with very specific functions and cellular localizations? Given its limited contribution in the mature epididymis, we hypothesized that it might be involved in the early epididymal differentiation and regionalization, and control gene expression in a temporal manner similar in other studies [15,52].

In conclusion, our study revealed a role of region-specific epididymal miRNAs, especially the miR-200 family in regulating epididymal regional gene expression in adult rats, which might contribute to the distinct physiological function in sperm maturation / storage of caput / cauda epididymis. On the other hand, although region-specific DNA methylation was detected in the mature epididymis, it didn’t correlate with the regional mRNA expression pattern. Epididymal DNA methylation might have function in the early developmental process rather than in the mature and fully differentiated state of the epididymis.

## Supporting Information

S1 Fig MeDIP-chip process and data verification. (A) Basic procedure for MeDIP and the following microarray. (B) Anatomic division of rat epididymides in our study. (C) Gel verification of genomic DNA sheared by sonication. (D) Confirmation of the MeDIP efficiency. (E) BSP verification of promoter DNA methylation status of 5 genes (Mbp, Slc7a1, Uox, Cdkl1 and Cdx2) in the rat epididymis. Although the ‘Peak score’ of each gene didn’t correlate with their actual promoter methylation levels, the MeDIP-chip data of the tested genes all agreed with the BSP results. (F) Promoter methylation status of the 5 genes detected by MeDIP-chip.
(TIF)

S2 Fig Regional difference of mRNA expression in rat epididymis. mRNA expression values (adapted from microarray data published by Jelinsky et al.,[31]) between 2 regions were displayed by scatter plot. Pearson correlation coefficient was used to characterize the difference.
(TIF)

S3 Fig Results of GSEA analysis performed on other candidate gene sets. For each graph: Left panel: enrichment score of a test gene set against their expression profile of caput and cauda epididymis. The x axis was the Cap/Cau level, and the y axis was the enrichment score of the test gene set. Right panel: heat map of test gene expression in caput and cauda region (Cap1-5 and Cau1-6 reflected the subdivision of each region in the study performed by Jelinsky et al., [31]). (A) Putative target genes of highly expressed miRNAs in the caput epididymis. (B) Putative target genes of highly expressed miRNAs in the cauda epididymis. (C) Genes with caput-specific promoter DNA methylation. (D) Genes with cauda-specific promoter DNA methylation.
(TIF)

S1 File Methods for BSP verification of MeDIP-chip data. (DOCX)

S1 Table MeDIP-chip results for rat epididymal regional DNA methylation. (XLS)

S2 Table GO enrichment of epididymal promoter methylated genes. (XLSX)

S3 Table miRNA microarray results for rat epididymal regional miRNA expression. (XLSX)

S4 Table GSEA analysis results of other candidate gene sets. (XLSX)

S5 Table GO enrichment of miR-200 family target genes. (XLSX)
