# Supplementary figures and images for: Epididymal Region-Specific miRNA Expression and DNA Methylation and Their Roles in Controlling Gene Expression in Rats
Source: PLoS One. 2015 Apr 22;10(4):e0124450. doi: 10.1371/journal.pone.0124450 (PMC4406618; doi:10.1371/journal.pone.0124450)

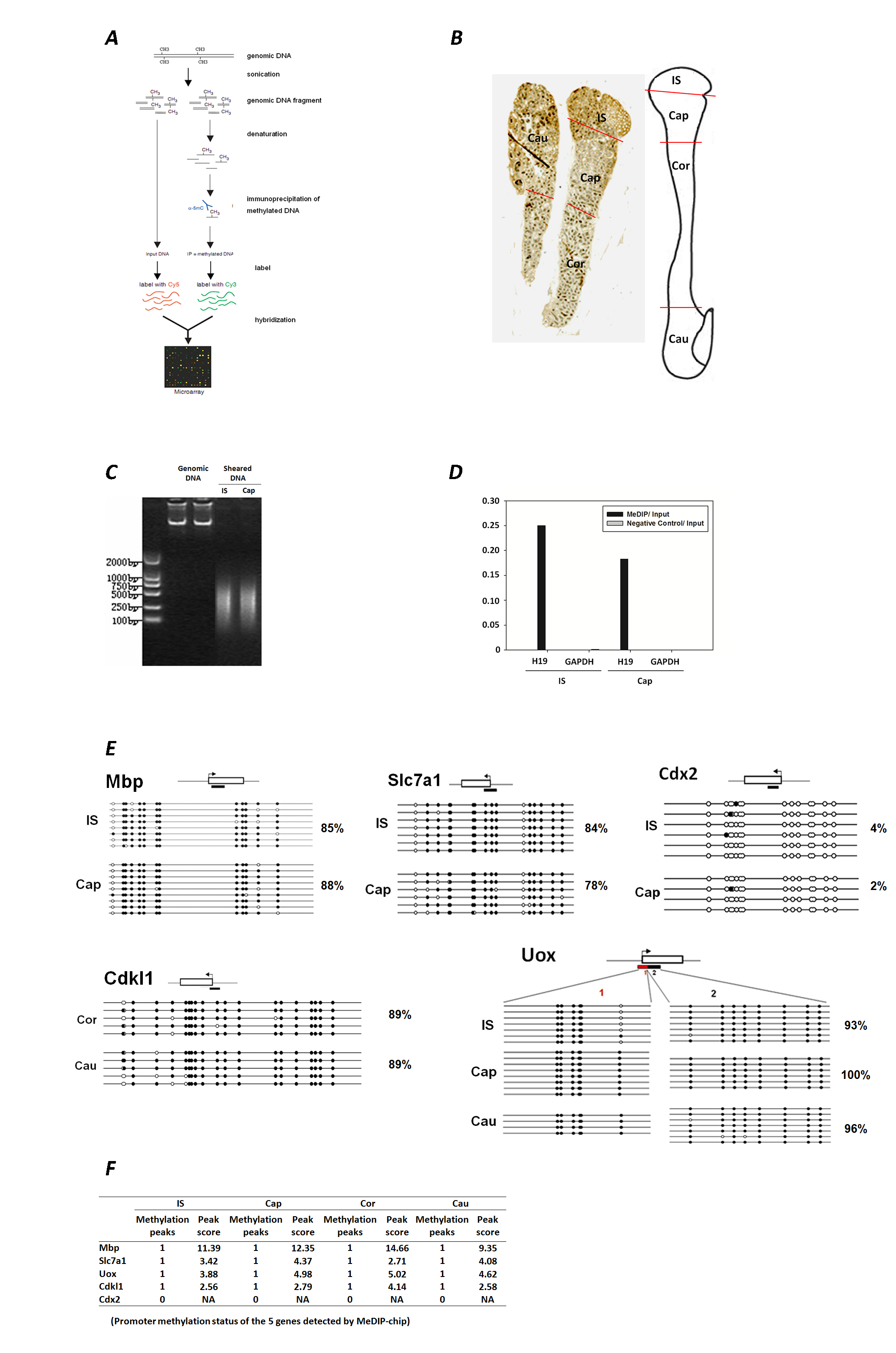

Supplement: S1 Fig — (A) Basic procedure for MeDIP and the following microarray. (B) Anatomic division of rat epididymides in our study. (C) Gel verification of genomic DNA sheared by sonication. (D) Confirmation of the MeDIP efficiency. (E) BSP verification of promoter DNA methylation status of 5 genes (Mbp, Slc7a1, Uox, Cdkl1 and Cdx2) in the rat epididymis. Although the ‘Peak score’ of each gene didn’t correlate with their actual promoter methylation levels, the MeDIP-chip data of the tested genes all agreed with the BSP results. (F) Promoter methylation status of the 5 genes detected by MeDIP-chip. (TIF) [file pone.0124450.s001.tif]

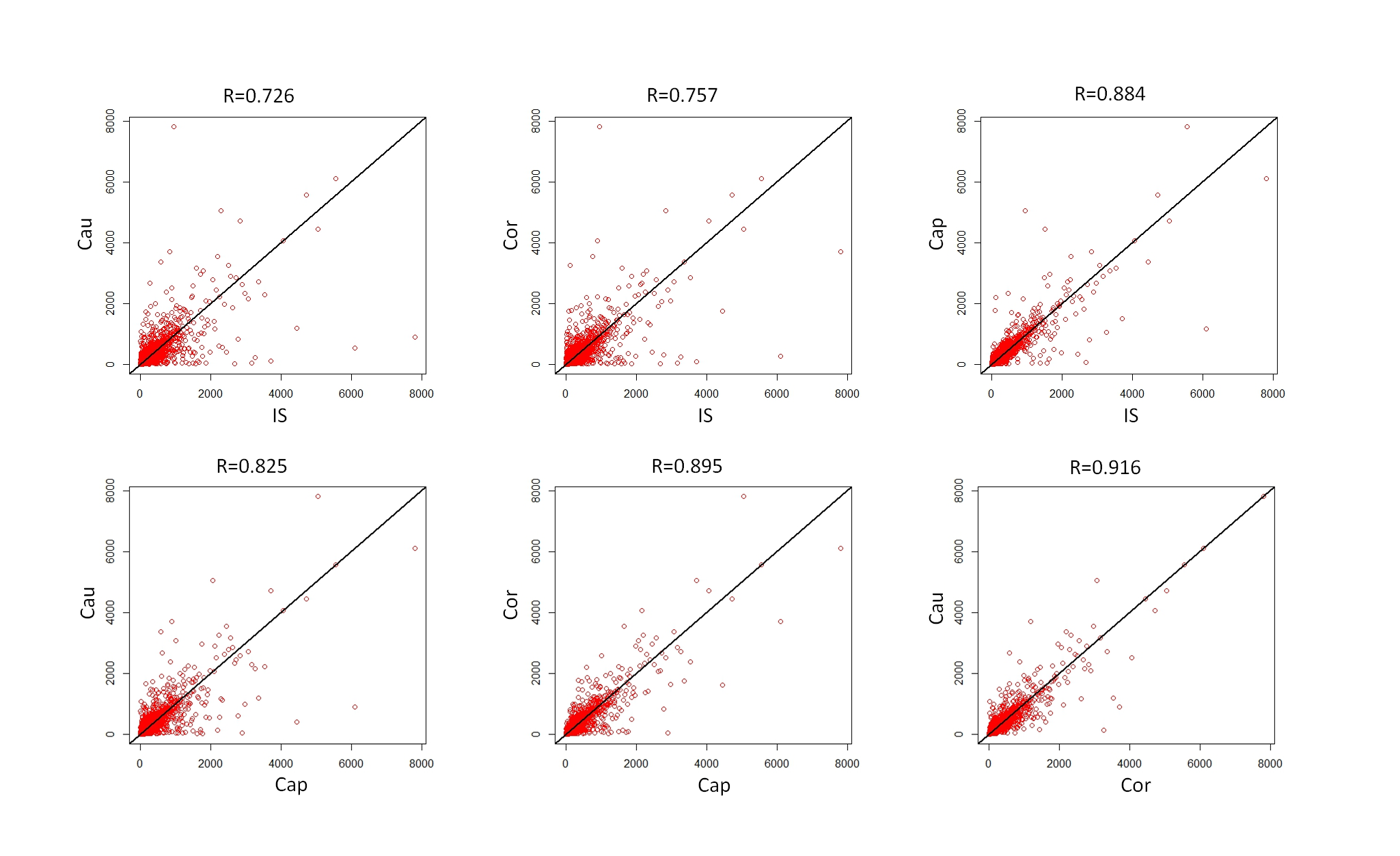

Supplement: S2 Fig — mRNA expression values (adapted from microarray data published by Jelinsky et al.,[31]) between 2 regions were displayed by scatter plot. Pearson correlation coefficient was used to characterize the difference. (TIF) [file pone.0124450.s002.tif]

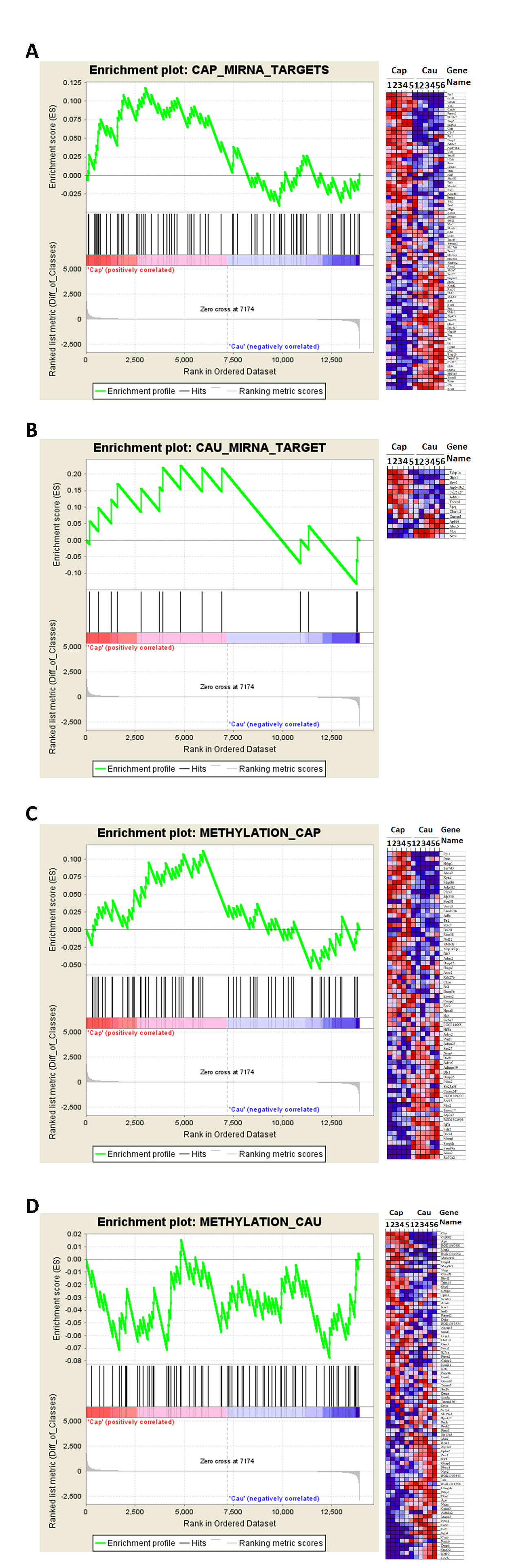

Supplement: S3 Fig — For each graph: Left panel: enrichment score of a test gene set against their expression profile of caput and cauda epididymis. The x axis was the Cap/Cau level, and the y axis was the enrichment score of the test gene set. Right panel: heat map of test gene expression in caput and cauda region (Cap1-5 and Cau1-6 reflected the subdivision of each region in the study performed by Jelinsky et al., [31]). (A) Putative target genes of highly expressed miRNAs in the caput epididymis. (B) Putative target genes of highly expressed miRNAs in the cauda epididymis. (C) Genes with caput-specific promoter DNA methylation. (D) Genes with cauda-specific promoter DNA methylation. (TIF) [file pone.0124450.s003.tif]
