# Supplementary material for: Epididymal Region-Specific miRNA Expression and DNA Methylation and Their Roles in Controlling Gene Expression in Rats
Source: PLoS One. 2015 Apr 22;10(4):e0124450. doi: 10.1371/journal.pone.0124450 (PMC4406618; doi:10.1371/journal.pone.0124450)
Supplement: S1 File — (DOCX) [file pone.0124450.s004.docx]

Bisulfite Sequencing PCR (BSP) verification of MeDIP-chip data

BSP approach was implemented to validate the methylation status of 5 gene promoters detected by MeDIP-chip. Briefly, 1 μg of genome DNA from each sample was bisulfite converted. Primers for BSP were designed by MethPrimer (http://www.urogene.org/methprimer/index1.html) [1] and listed below. The bisulfite conversion products were amplified using the following PCR program: 94℃ for 4 min, followed by 35 cycles of 94℃ for 30 sec, 55℃ for 30 sec, 72℃ for 30 sec and then extension at 72℃ for 10 min. Then, bisulfite PCR products were cloned into a T vector (TaKaRa, Japan) after purification, and 30 clones were selected for further Sanger sequencing for each gene. The promoter methylation status of Mbp, Slc7a1, Uox, Cdkl1 and Cdx2 were examined by BSP.

| **Primers for BSP** | **Forward (5' to 3')** | **Reverse (5' to 3')** |
| --- | --- | --- |
| Mbp | GGGGTGTTTATTTAGTTGATTTAGG | ATAATACCATCACATCCAAACCTTC |
| Slc7a1 | TTGGGTTAGTGGTGTTTAATTTTTT | AAATCATAACCCCTTTACAAATCAC |
| Uox-1 | TTTTTGTTGAGATGATATGTGTGTGT | TCTATAATAACCCCCAAAAAAACCT |
| Uox-2 | GAGGTTTTTTTGGGGGTTATTATAG | ACAAAAAATAACAACCCAACCTCTA |
| Cdkl1 | GTAGGGTTAGGGATTTTGTTAGGTT | AATCTCATTTCCCTCTAAAAAAAA |
| Cdx2 | TAAAGTGTATTTAGGTTGGAAGGAG | AACACAAACACCAATAACTAAAAAC |

**References:**

1. Li LC, Dahiya R (2002) MethPrimer: designing primers for methylation PCRs. Bioinformatics 18: 1427-1431.
